# Anthropogenic Transmission of SARS-CoV-2 from Humans to Lions, Singapore, 2021

**DOI:** 10.3201/eid2912.221916

**Published:** 2023-12

**Authors:** Yin Cheong Aden Ip, Adrian Tan, Jasmine Ong, Charlene Judith Fernandez, Clara Lau, Wai Kwan Wong, Siow Foong Chang, Him Hoo Yap, Kenneth B.H. Er

**Affiliations:** National Parks Board, Singapore

**Keywords:** COVID-19, SARS-CoV-2, viruses, respiratory infections, zoonoses, Delta variant, coronavirus, reverse zoonosis, human-to-animal transmission, *Panthera*, lion, zoo, nanopore sequencing, viral genome, endangered species, Singapore

## Abstract

In Singapore, 10 captive lions tested positive for SARS-CoV-2 by real-time PCR. Genomic analyses of nanopore sequencing confirmed human-to-animal transmission of the SARS-CoV-2 Delta variant. Viral genomes from the lions and zookeeper shared a unique spike protein substitution, S:A1016V. Widespread SARS-CoV-2 transmission among humans can increase the likelihood of anthroponosis.

We investigated natural SARS-CoV-2 infection in captive African (*Panthera leo*) and Asiatic (*Panthera leo persica*) lions at a zoo in Singapore during increased Delta variant community infections. Understanding virus dynamics in different hosts is crucial for preventing interspecies transmission and protecting endangered species (*1*,*2*).

We studied 14 lions, 9 Asiatic and 5 African, that were housed in separate enclosures. On November 6, 2021, respiratory signs developed in a male Asiatic lion (AS-M1) (Appendix 1). On November 7, three Asiatic lionesses (AS-F1, AS-F2, and AS-F3) in the same enclosure exhibited similar clinical signs. A male African lion (AF-M3) in a separate enclosure developed clinical signs on November 8.

Eighteen zookeepers cared for and had close (within ≈1 m) but not direct contact with the lions. Six zookeepers tested COVID-19–positive beginning November 1, 2021, and 4 experienced mild respiratory symptoms starting on November 2. 

To minimize stress on the animals, only 2 lions that had more severe signs, AS-M1 and AS-F1, were anesthetized for nasal and oropharyngeal sample collection on November 8. On November 9, we confirmed SARS-CoV-2 infection in the lions by real-time reverse transcription PCR (rRT-PCR); cycle quantitation (Cq) values were <40. Nasal swab samples from AS-M1 and AS-F1 had the highest viral loads (Cq 23.05 for AS-M1, 24.47 AS-F1). We conducted noninvasive infection monitoring for 3 weeks by collecting and testing individual and pooled fecal samples from both lion enclosures. AF-M3 had the highest fecal sample viral load, Cq 36.02. 

Within 5 days of the index case, 10 lions (all 9 Asiatic and 1 African) were infected. Most (8/10) clinically recovered from respiratory signs within 2 weeks; 2 lions took longer to recover, but all animals had recovered by December 3, 2021. Full recovery in the lions was determined by low viral RNA loads (Cq >40), absence of clinical signs, and resumption of normal behavior.

We sequenced RNA from nasal swab samples of AS-M1 and AS-F1 and 1 fecal sample from AF-M3 on the MinION R9.4.1 (Oxford Nanopore Technology, https://nanoporetech.com) platform using ARCTIC-CoV V1/V3 protocols (J.R. Tyson et al., unpub. data, https://doi.org/10.1101/2020.09.04.283077). The 3GS analysis pipeline from Genome Detective (*3*) generated preliminary contigs, which we stitched together by using sequence alignment information from a zookeeper’s publicly available SARS-CoV-2 sequence (GISAID accession no. EPI_ISL_6600690; https://www.gisaid.org). We assessed the assembled sequences by using NextClade (*4*) to identify mutations and frameshifts compared with a wild-type reference sequence (GenBank accession no. NC_045512.2) and subsequently corrected alignment artifacts in the bam file (Appendix 2).

We assembled 2 complete SARS-CoV-2 genomes (GenBank accession nos. OP393893.1 and OL677176.2) from nasal swab samples collected from AS-M1 and AS-F1. We obtained a partial genome assembly from a fecal sample from AF-M3 but did not analyze it further. 

We conducted a phylogenomic analysis on 39 complete viral genomes, comprising 2 genomes from lions in this study and 37 sequences from GISAID, including the zookeeper’s sequence. We built a maximum-likelihood tree by using RAxML-ng version 1.1.0 (https://github.com/stamatak/standard-RAxML) with 2,000 bootstrap replicates and used the wild-type reference sequence as the outgroup. The tree revealed that sequences from the zookeeper and Asiatic lions nested within the same subclade (Figure). Those findings and the high (99.98%) viral genetic similarity between the lions and zookeeper strongly suggest that SARS-CoV-2 infection in the lions occurred through a human-to-animal (anthropogenic) transmission route.

**Figure:**
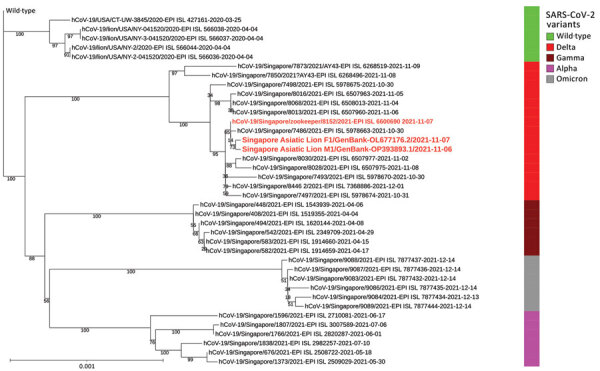
Maximum-likelihood phylogenomic tree from a case of anthropogenic transmission of SARS-CoV-2 from humans to lions, Singapore, 2021. Tree reconstructed from sequences of 2 lions and 1 zookeeper (red bold text), along with 36 other publicly available sequences representing 4 variants of concern from Singapore, cases of infected lions from the Bronx Zoo, and the wild-type reference genome (GenBank accession no. NC_045512.2) as the outgroup. Scale bar indicates nucleotide substitutions per site. EPI, GISAID (https://www.gisaid.com) EpiFlu database.

We used Pangolin (https://github.com/cov-lineages/pangolin) to identify the subclade as Delta lineage AY.23.1, consistent with the predominant circulating strain in Singapore at that time. Both lions’ sequences had 10 key Delta variant spike protein mutations and 2 open reading frame 8 amino acid deletions at positions D119- and F120-, compared with sequences from cases in Singapore (Appendix 1) (*5*). The lions, zookeeper, and 1 community case shared a unique spike protein mutation (S:A1016V), suggesting a potential founder’s effect from this anthroponotic transmission event. Our investigation determined that the zookeepers were likely infected 6 days before the lion index case. The lions were not vaccinated against SARS-CoV-2, but 94% of the population of Singapore was fully vaccinated by November 2021.

This study highlights the vulnerability of captive and endangered animal populations to SARS-CoV-2 transmission from humans (*5*–*8*). Close contact between zookeepers and the lions likely led to the transmission, emphasizing the crucial need for strict infection control measures in captive animal facilities, especially during periods of increased community transmission of viruses (*7*).

The implications of SARS-CoV-2 infection in captive lions extend beyond animal health and welfare and can have consequences for the conservation of protected species. Insights from studies on minks and hamsters shed light on the potential for animal-to-human transmission (*6*,*8*). However, mass culling, as noted in those studies of small mammals, is an impractical approach for large or endangered animal species.

Lions already face numerous threats, including habitat loss, poaching, and disease; introduction of a novel virus like SARS-CoV-2 could have devastating consequences for their populations (*7*). Therefore, strengthening biosecurity measures in wildlife conservation centers and promoting vaccination of susceptible animal species whenever feasible and safe are crucial for mitigating viral transmission and protecting vulnerable wildlife populations (*1*,*9*).

Appendix 1 Additional information on anthropogenic transmission of SARS-CoV-2 from humans to lions, Singapore, 2021.

Appendix 2 Genomic information from anthropogenic transmission of SARS-CoV-2 from humans to lions, Singapore, 2021.
