# Supplementary material for: Anthropogenic Transmission of SARS-CoV-2 from Humans to Lions, Singapore, 2021
Source: Emerg Infect Dis. 2023 Dec;29(12):2550–3. doi: 10.3201/eid2912.221916 (PMC10683833; doi:10.3201/eid2912.221916)

Article DOI: https://doi.org/10.3201/eid2912.221916

EID cannot ensure accessibility for supplementary materials supplied by authors. Readers who have difficulty accessing supplementary content should contact the authors for assistance.

Anthropogenic Transmission of SARS-CoV-2 from Humans to Lions, Singapore, 2021

Appendix 1

Wet Laboratory and Phylogenomic Analyses Methods

Viral RNA Extraction and RT-qPCR Amplification

The swab samples were placed in virus transport media while blood samples were added to tubes with ethylenediaminetetraacetic acid (EDTA) and transported on ice to CAVS for laboratory diagnostic investigation. Upon receipt of samples, nasal and oropharyngeal swabs were vortexed for 15 s and placed in 1.5 mL eppendorf tubes for ribonucleic acid (RNA) extraction. Fecal samples were diluted 1:10 in virus transport media (VTM), vortexed for 15 s, pulse centrifuged and the decant was used for RNA extractions. EDTA blood samples were processed directly. Viral RNA was extracted using QIAGEN IndiMag Pathogen Kit on an automated nucleic acid extraction machine Applied Biosystems MagMax Total Nucleic Acid Extraction System using manufacturer’s protocols and eluted in 100 µL of elution buffer.

Preliminary detection of SARS-CoV-2 from RNA extracts was ascertained with three assays, (i) screening (E assay for betacoronavirus), (ii) confirmatory (C assay for SARSCoV) and (iii) discriminatory (D assay for SARS-CoV-2) with real-time reverse transcription polymerase chain reaction assays (rRT-PCR) on the Applied Biosystems 7900 Fast. All three rRT-PCR assays were done in a total volume of 25 µL reaction mixture, consisting of 12.5µL of 2´ RT-PCR buffer (Applied Biosystems AgPath-ID One Step RT-PCR reagents), 1µL of 25´ RT-PCR enzyme mix, 2.5 µL of RNA template, with varying volumes in primers and probes: (i) E assay: 1 µL (10 µmol) each of forward: E-Sarbeco-F1 ACAGGTACGTTAATAGTTAATAGCGT and reverse: E-Sarbeco-R2 ATATTGCAGCAGTACGCACACA primers, 1 µL (6 µmol) of probe: E-Sarbeco-P1

FAMACACTAGCCATCCTTACTGCGCTTCGBBQ, and 6 µL of nuclease-free water; (ii, iii) D and C assays: 1.5 µL (10 µmol) of forward: RdRp-SARSr-F2 GTGARATGGTCATGTGTGGCGG and 2 µL of reverse: RdRp-SARS-R1 CARATGTTAAASACACTATTAGCATA, 1 µL (6 µmol) of probe: RdRp-SARSr-P2 FAM-CAGGTGGAACCTCATCAGGAGATGCBBQ, and additional probe RdRp-SARSr-P1 FAM-CCAGGTGGWACRTCATCMGGTGATGCBBQ for C assay, and finally 4.5 µL and 3.5 µL of water for D and C assays respectively. The thermal cycling profile consisted of an initial reverse transcription step at 45°C for 10 minutes, an initial enzyme activation step at 95°C for 10 minutes, followed by 45 cycles of 94°C for 15 seconds and 58°C for 30 seconds. Samples that returned a Cq value of <42 were considered as a detection of SARSCoV-2 nucleic acid material.

Multiplex PCR and Oxford Nanopore Sequencing and Assembly of SARS-CoV-2 Genomes

Two RNA samples from nasal swabs of two Asiatic lions (Cq = 23.05 and 24.47) and one individual fecal sample from an African lion (Cq = 36.02) were sequenced on the MinION Nanopore platform. One of the nasal swab samples (viral inactivated at 56 degrees for 2 hours) was re-extracted using QIAamp Viral RNA extraction kit following manufacturer's instructions. All post-PCR, reverse transcription, and library purification steps were done with KAPA pure beads (Roche Sequencing).

Nasal swab RNA was converted directly to double-stranded complementary DNA (cDNA) by first-strand synthesis with the SuperScript III kit (Thermo Fisher Scientific) containing random hexamers. Briefly, 1 µL each of dNTPs and random hexamers (50 µmol, Invitrogen) were added to 11 µL of template RNA and incubated at 65°C for 5 minutes before placing on ice for 1 minute. Next, 1 µL of 0.1M DTT, 4 µL of 5′ First Strand buffer, 1 µL of RNaseOUT (Invitrogen) and 1 µL of SuperScript III were added to each cDNA conversion reaction assay of the previous step. The final 20 µL mix was incubated at 42°C for 50 minutes, followed by 70°C for 10 minutes, and placed on ice for 5 minutes. The PCR reaction products were purified, and cDNA was stored at −20°C until library preparation.

Separately, the single fecal sample underwent a Sequence-Independent Single Primer (SISPA) enrichment (*1*) to increase nucleic acid yield for whole genome sequencing (*2*,*3*). Briefly, first-strand cDNA was synthesized in a 20 µL reaction consisting of 7 µL of viral RNA

from fecal samples, 50 pmol of 10 μmol primer A (5′- GTTTCCCACTGGAGGATA-NNNNNNNNN- 3′), SuperScript III

Reverse transcription (Thermo Fisher Scientific) and dNTPs (10 μmol), with incubation at 25°C for 5 minutes, followed by 50°C for 60 minutes and 70°C for 15 minutes. Second strand synthesis continued in the 20 μL cDNA mix forming a total volume of 24 μL with 2.5 μL Klenow buffer, 10 pmol Primer A, and dNTPs (1 μmol), with one-hour incubation at 37°C. The doublestranded cDNA was purified, and sequence-independent PCR amplification was done in a final reaction volume of 50 μL containing 2× LongAmp® Taq Master Mix (NEB), 50 pmol of 10 μmol primer B (5′- GTTTCCCACTGGAGGATA-3′). The PCR cycling was performed as follows: 94°C for 30 s, followed by 30 cycles of 94°C for 15 s, 50°C for 30 s and 65°C for 5 minutes, with a final extension at 65°C for 10 minutes, followed by purification of PCR products.

Multiplex PCR was used to enrich viral cDNA of all three samples using Q5 Hot-start HF polymerase with ARCTIC-CoV V1 and V3 protocols (J.R. Tyson et al., unpub. data, https://doi.org/10.1101/2020.09.04.283077), where the tiling primer schemes targeted SARS-CoV-2 genome-wide amplicons of 400-bp in length. PCR thermal cycling profiles consisted of 98°C at 30 seconds, followed by 25 (Cq <25 nasal swab samples) or 35 cycles (Cq: 36.02 fecal samples) of 98°C for 15 seconds and 65°C for 5 minutes, before a final placement on ice for 5 minutes. Forward and reverse primer PCR reactions (odd and even primer pools) were conducted separately and pooled immediately after PCR, purified, and end-repaired with the NEB Ultra II companion kit.

MinION nanopore libraries were prepared using the Native Barcoding (EXP-NBD104) and ligation sequencing (SQK-LSK109) kits (Oxford Nanopore Technologies, Oxford, UK). Finalized cDNA libraries of 8–12 ng were loaded on three Flo-MIN106 R9.4 flow cells and sequenced for 22–36 hours.

Phylogenomic Analyses

MAFFT v. 7.490 to generate multiple sequence alignments for the 39 sequences intended for tree inference (Figure in main article text). The final alignment only consisted of coding regions and stop codons were verified to be present only at the terminal end of each gene. Next, RaxML-ng v1.1.0 was used to infer a maximum-likelihood tree, using the GTR + I + G model of

nucleotide substitution with 2000 bootstrap replicates, and the Wuhan-Hu-1 reference sequence (GenBank: NC_045512.2 / GISAID: EPI_ISL_402119) was used as the outgroup. Convergence of trees were attained at 1950 bootstraps. Inferred trees were visualized and annotated on Interactive Tree of Life v5 (iTOL) (*4*).

Bioinformatic Analyses

Genome Detective Assembly of Nanopore Reads.

Consensus sequences were generated by Genome Detective using a consensus algorithm that is reference based for 3GS reads (J.R. Tyson et al., unpub. data, https://doi.org/10.1101/2020.09.04.283077). For nasal swab samples from AS-M1 and AS-F1, the consensus viral sequences consisted of 29,822 bp, had mean read depths of 364.99 and 1,050.59, median read depths of 199 and 466, read depth ranges of 2–3,013 and 1–11,918, and mean base quality scores of 19.1 and 19.3.

Long reads are blasted to candidate reference sequences (NT&AA), aligned with aga (a combined NT&AA aligner, https://www.genomedetective.com/app/aga) to the top candidates, the consensus sequences are then constructed. Due to aligning in the NT and AA domain simultaneously, aga is able to detect and correctly handle frame shifts, and also corrects errors in monomer repeat regions as those errors are typical for nanopore sequencers.

Stitching of Sequences

The consensus contigs obtained from Genome Detective were aligned to the Singapore reference SARS-CoV2 sequence (EPI_ISL_6600690). Based on this reference, the gaps were determined. Passed nanopore reads (Q-score $\geq$8) were mapped using Minimap2 to this reference and the consensus bases (Base Quality $\geq$10) in the reads that encompassed these gaps were used to stitch the contigs together. The consensus cut off was at 80%, but when the cut off cannot be achieved, an N base was used as a fill in. No explicit depth cut off filter was applied in this analysis. Instead, the depth filter is implicitly defined with a consensus 80% cut off as demonstrated in Appendix 1 Table 1.

Appendix 1 Table 1. Relationship between depth and consensus for SARS-CoV-2 sequences

Depth	Minimum consensus bases	% Effective consensus
1	1	100
2	2	100
3	3	100
4	4	100
5	4	80
6	5	83
7	6	86
≥6	...	...

Thus, the consensus cut off is more stringent for lower depths as seen in the table. Insertions and deletions were not encountered during the stitching process.

The rationale for this approach is that our sequencing runs included sequence fragments that were not reliably amplified by the ARCTIC V1 and V3 primers. These regions would have low read depth and thus were filtered out during the consensus building process.

0.51% and 2.4% of the nucleotides for AS-F1 and AS-M1 sequences were added during this process. Note that for a portion of AS-F1, the read depths were high but that region was not included in the consensus sequence generated by Genome Detective (Appendix 1 Table 2). The script for stitching is available at https://github.com/atks/cavs/blob/master/var/analysis/stitch_contigs.py.

Appendix 1 Table 2. Consensus sequences generated by Genome Detective*

Sequence	Length, bp	No. contigs to stitch	Added sequences (N bases)	Mean depth	Gap depths	Gap added bases
AS-F1	29825	2	153 (2Ns)	451.14	1475.96, 8.00, 15.05	46, 68, 39
AS-M1	29861	4	706 (30Ns)	11.7	1.77, 6.66, 7.75, 22.3, 9.55	13, 252, 179, 213, 49

*bp, base pairs.

Manual Polishing of AS-F1 Stitched Sequence

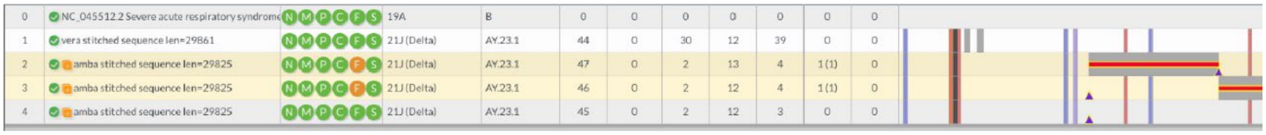

Appendix 1 Figure 1. NextClade Output showing mutations in this stud's viral genomes when compared with the reference genome.

The mutations detected by NextClade (Appendix 1 Figure 1) were examined in the mapped reads to remove any artifacts introduced by Genome Detective. These changes are summarized in Appendix 1 Figures 2–5.

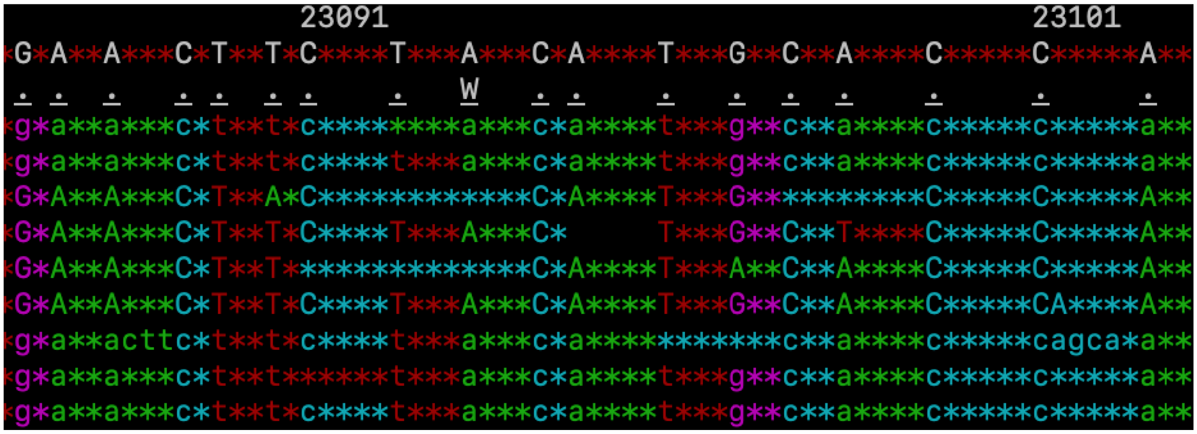

Appendix 1 Figure 2. Position 23093–23095 consisting of 2 populations of haplotypes – ACAT and TCAT. The sequence from Genome Detective has TAC in place that induces a frameshift. Changing TAC >ACAT removes the frameshift but introduces an inserted Proline CCT and a downstream frameshift.

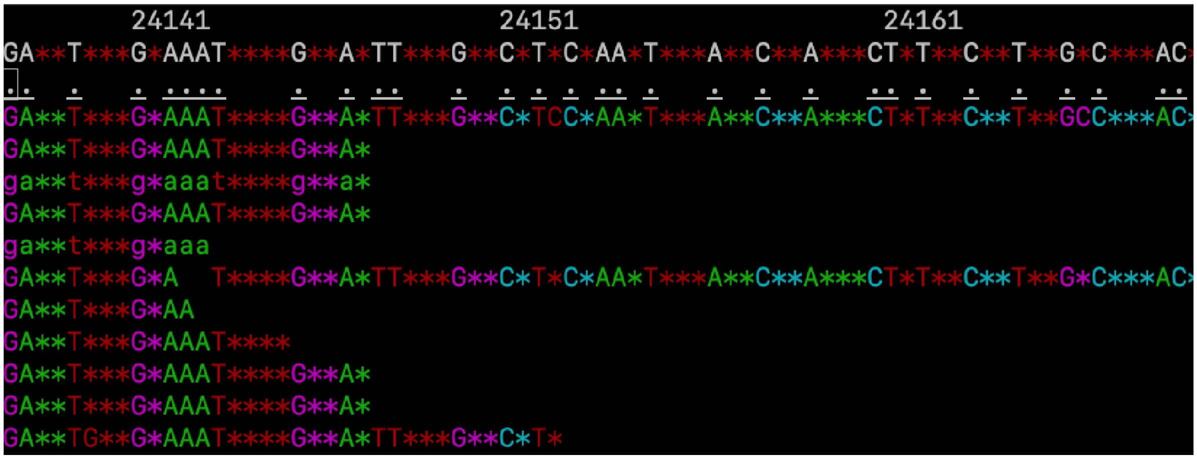

Appendix 1 Figure 3. Position 24151–24154. The sequence from Genome Detective has GGTG, this is detected at a position where Minimap2 soft clips many reads. There are nonetheless reads that give support to show that the reference sequences are largely conserved. Position 23093 has two populations of reads with A and T bases. We replaced GGTG with TTG due to the reads supporting the reference. Frameshift has been removed.

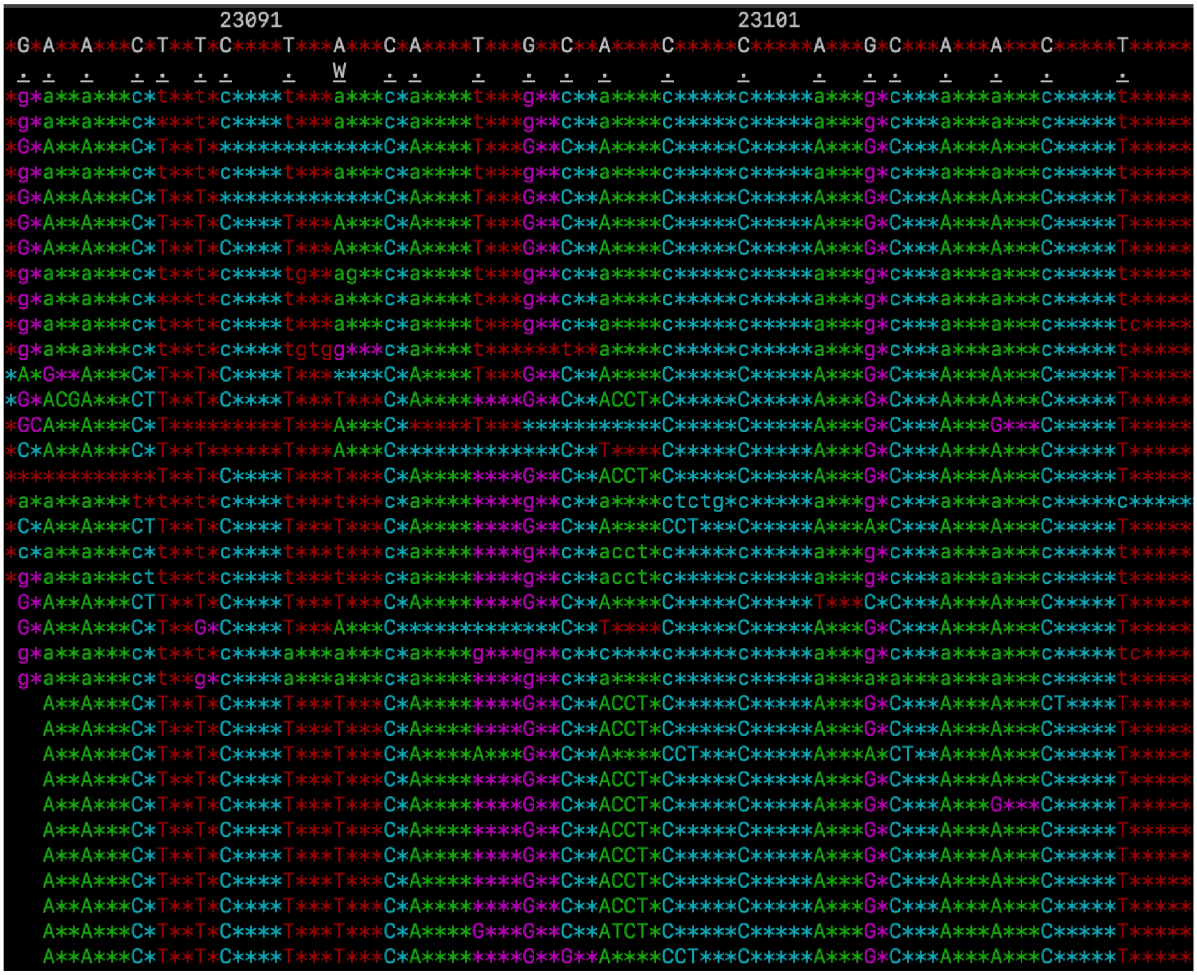

Appendix 1 Figure 4. Positions 23100–23102 contain the inserted CCT proline codon. Once again, there are two populations of reads. However, the CCT sequence tends to occur on the tail end of reads and accompanies the T base variant at position 23093. We defer again to the reads that support the reference sequence. CCT has been deleted.

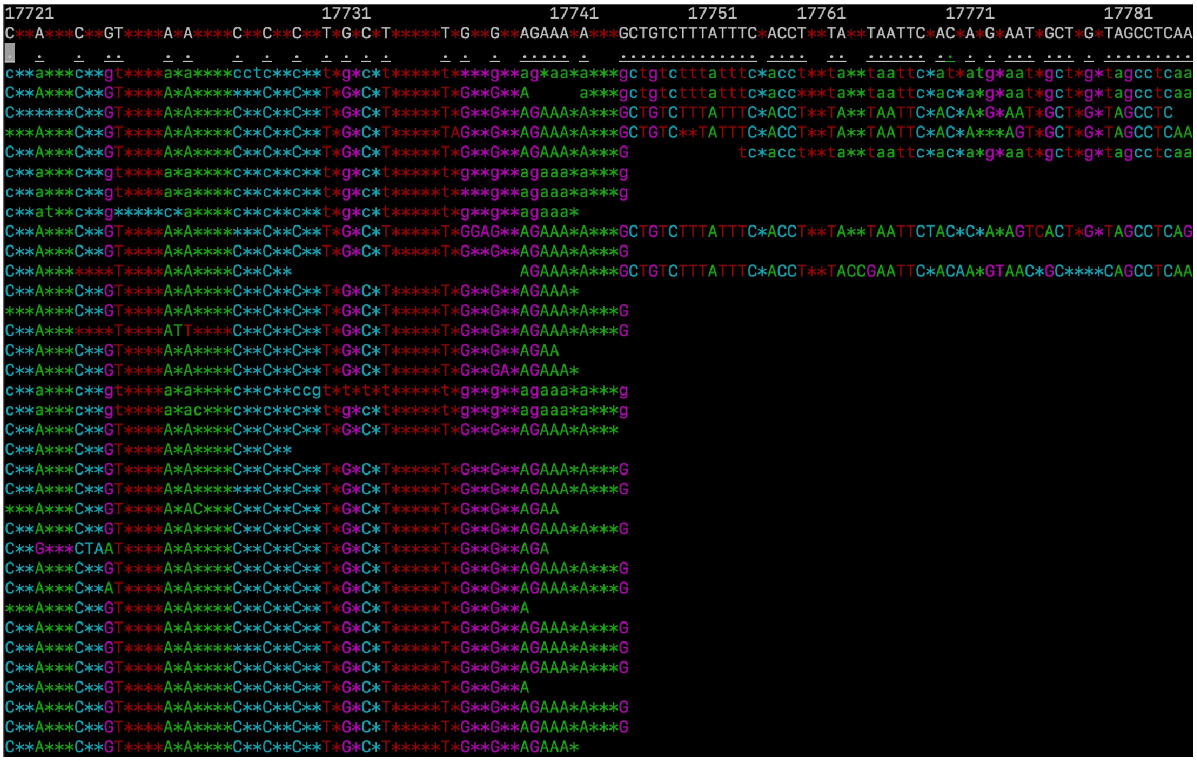

Appendix 1 Figure 5. Position 17762 is reported to be an A missense mutation in Genome Detective's sequence. The allele, however, occurs on the soft clipped portion of many Minimap2 reads. There are reads that support the reference, thus we changed the base from A to C.

Manual Polishing of AS-M1 Stitched Sequence

The mutations detected by NextClade were examined in the mapped reads to remove any artifacts introduced by Genome Detective. These changes are summarized in Appendix 1 Figure 6.

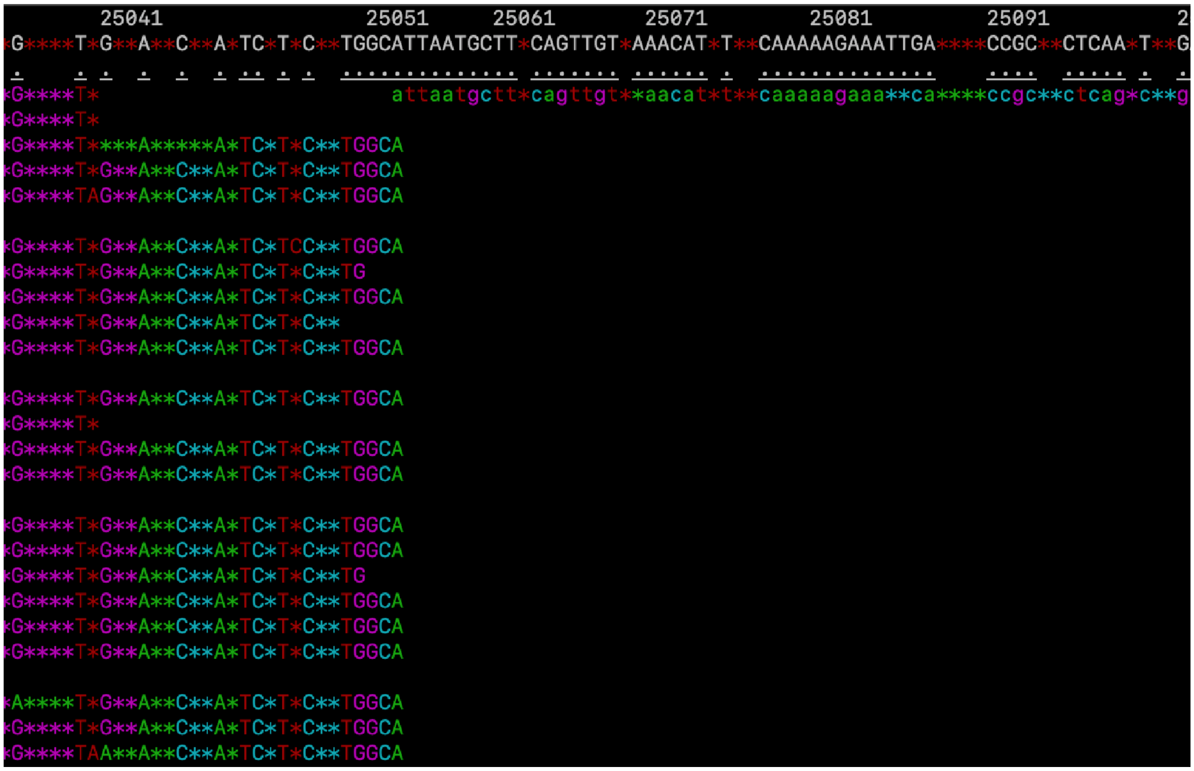

Appendix 1 Figure 6. Position 25054–25092. Large 13 AA insert appears to be soft clipped on Minimap2 alignments. AGGTGCTGCAGGTAGAAAGAAGCAGAATCGGATTAACCC = >delete.

Coverage of Arctic V1 and V3 Primers

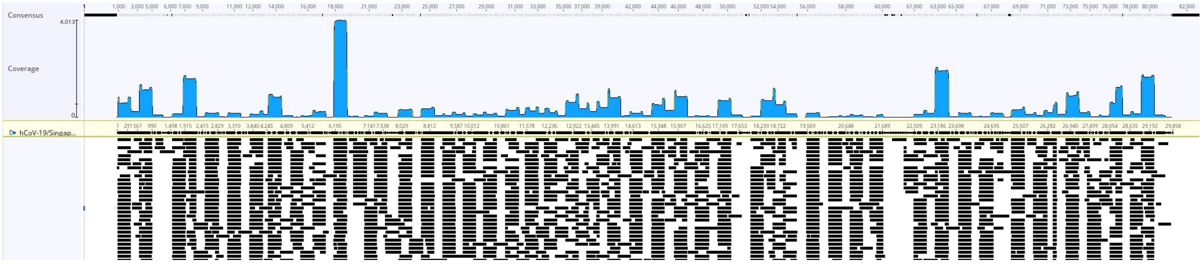

Appendix 1 Figure 7. Coverage of Arctic V1 and V3 primers for samples from Asiatic lion AS-F1.

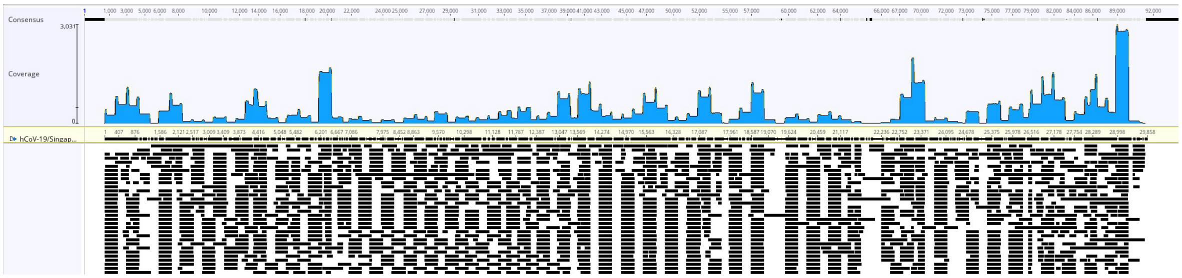

Appendix 1 Figure 8. Coverage of Arctic V1 and V3 primers for samples from Asiatic lion AS-M1.

References

1. Chrzastek K, Lee DH, Smith D, Sharma P, Suarez DL, Pantin-Jackwood M, et al. Use of sequence-independent, single-primer-amplification (SISPA) for rapid detection, identification, and characterization of avian RNA viruses. Virology. 2017;509:159–66. PubMed https://doi.org/10.1016/j.virol.2017.06.019

2. Fernández-Bellon H, Rodon J, Fernández-Bastit L, Almagro V, Padilla-Solé P, Lorca-Oró C, et al. Monitoring natural SARS-CoV-2 infection in lions (*Panthera leo*) at the Barcelona Zoo: viral dynamics and host responses. Viruses. 2021;13:1683. PubMed https://doi.org/10.3390/v13091683

3. Mishra A, Kumar N, Bhatia S, Aasdev A, Kanniappan S, Sekhar AT, et al. SARS-CoV-2 Delta variant among Asiatic lions, India. Emerg Infect Dis. 2021;27:2723–5. PubMed https://doi.org/10.3201/eid2710.211500

4. Letunic I, Bork P. Interactive Tree Of Life (iTOL) v5: an online tool for phylogenetic tree display and annotation. Nucleic Acids Res. 2021;49:W293–96. PubMed https://doi.org/10.1093/nar/gkab301

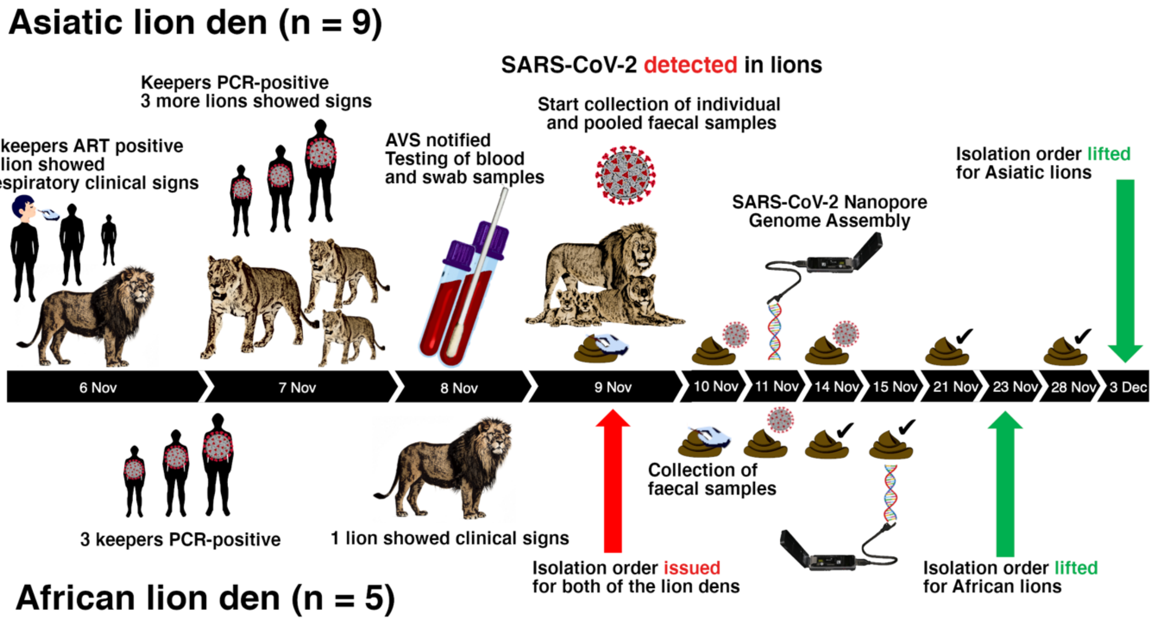

Appendix 1 Figure 9. Timeline of events providing an overall graphical summary of SARS-CoV-2 infections of lions and zookeepers at the MWG, and the subsequent diagnostic efforts by CAVS for confirmation and surveillance of infection up till recovery.

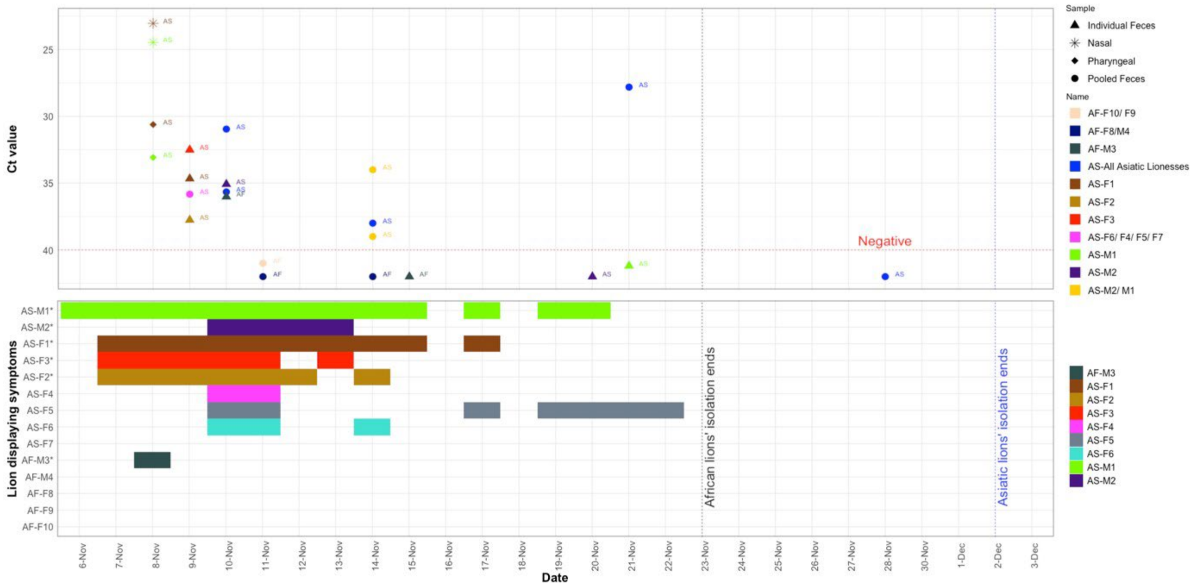

Appendix 1 Figure 10. Tracking viral RNA detection trends of all naturally infected lions (colors) using rRT-PCR on nasal swab, oropharyngeal swab, and fecal samples; represented by symbols (upper panel) and are largely congruent with the recorded clinical observations of signs (lower panel). African and Asiatic lions are indicated by codes "AF" and "AS" in the legends and data points, respectively.

Appendix 1 Figure 11 (following page). Maximum likelihood tree inferred from Singapore's community cases during October 25–November 23, 2021. The tree consists of 1,003 genomes that include the wildtype as outgroup, zookeeper sequence, and M1 and F1 sequences from lion cases.

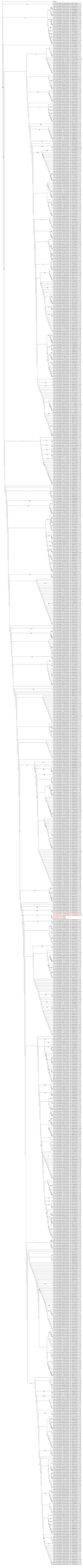

Supplement: Appendix 1 — Additional information on anthropogenic transmission of SARS-CoV-2 from humans to lions, Singapore, 2021. [file 22-1916-Techapp-s1.pdf]
